# Evaluation of the Shear Bond Strength of Four Bioceramic Materials with Different Restorative Materials and Timings

**DOI:** 10.3390/ma15134668

**Published:** 2022-07-03

**Authors:** Abeer S. Alqahtani, Ayman M. Sulimany, Abdullah S. Alayad, Abdulaziz S. Alqahtani, Omar A. Bawazir

**Affiliations:** 1 Department of Pediatric Dentistry and Orthodontics, College of Dentistry, King Saud University, Riyadh 11545, Saudi Arabia; asulimany@ksu.edu.sa (A.M.S.); obawazir@ksu.edu.sa (O.A.B.); 2 Department of Restorative Dental Science, College of Dentistry, King Saud University, Riyadh 11545, Saudi Arabia; alayad@ksu.edu.sa; 3 Department of Prosthetic Dental Science, College of Dentistry, King Saud University, Riyadh 11545, Saudi Arabia; absalqahtani@ksu.edu.sa

**Keywords:** bioceramic, mineral trioxide aggregate, NeoPUTTY, NeoMTA2, premixed bioceramics, resin composite, resin-modified glass ionomer

## Abstract

The objectives of this study were to evaluate the shear bond strength (SBS) of a resin composite (RC) and a resin-modified glass ionomer (RMGI) to four different bioceramic materials and to compare the effects of the immediate vs. delayed placement of restoration on the SBS. A total of 160 Teflon blocks and 40 blocks/material, were randomly filled with one of the bioceramic materials (NeoPUTTY^®^, NeoMTA2^®^, TotalFill^®^ BC RRM™ Fast Set Putty, and ProRoot^®^ MTA). The restoration was performed immediately or in a delayed time frame (after 7 days) using a Filtek™ Z350 XT Flowable composite (bonded to the bioceramic materials using Single bond universal 3M) or GC Fuji II LC^®^ RMGI. The SBS test was performed at a crosshead speed of 1 mm/min, and the failure mode was evaluated under a digital microscope by one blinded examiner. One-way analysis of variance (ANOVA) with the Games–Howell post hoc test was used to compare the mean SBS between the groups. The mean SBS of the bioceramic materials to RC was significantly higher than to RMGI except for ProRoot MTA (*p*-value 0.65). The SBS values to RC were as follows: ProRoot MTA (7.64 MPa); NeoMTA2 (8.57 MPa) which was significantly higher than both NeoPUTTY (4.04 MPa) and TotalFill^®^ BC RRM™ Fast Set Putty (4.38 MPa). For RMGI groups, ProRoot MTA showed the highest SBS (7.18 MPa), followed by NeoMTA2 (4.15 MPa), NeoPUTTY (1.62 MPa), and TotalFill^®^ BC RRM™ Fast Set Putty (1.54 MPa). The delayed timing restoration showed a significantly higher SBS than the immediate, except for the immediate RMGI restoration with MTA. To conclude, the SBS of RC to the bioceramic materials was significantly higher than RMGI, except for ProRoot MTA. Both restorative materials had a significantly higher SBS to the MTA groups in comparison to premixed bioceramics. Delayed RC restoration had a higher SBS than immediate restoration. Similarly, delayed RMGI restoration had a higher SBS than immediate restoration with premixed bioceramic but not with MTA.

## 1. Introduction

Nowadays, a variety of bioceramic materials are used in the vital pulp therapy (VPT) of primary teeth [1]. Among these bioceramic materials, Mineral Trioxide Aggregate (MTA) is the most widely used in VPT because of its superior physical and chemical properties and its high success rate as reported in multiple systematic reviews and meta-analyses [2,3,4,5,6,7,8]. Despite having superior properties, the staining potential and difficult handling of MTA are considered the major drawbacks [9,10,11,12,13,14,15]. To overcome these drawbacks, several manufacturers have produced newer versions of stain-free MTA and premixed bioceramics.

NeoMTA 2 (Nusmile Inc., Houston, TX; USA) is a stain-free MTA where tantalum oxide is added as a radiopacifying agent instead of bismuth oxide, which is the cause of discoloration especially when it comes in contact with sodium hypochlorite [9,16]. NeoMTA 2 is a modified version of NeoMTA plus, which was proven to be stain-free in laboratory studies and has a success rate similar to MTA in primary molar pulpotomies [16,17,18]. The premixed bioceramics were first introduced in 2007 by a Canadian company as iRoot SP injectable root canal sealer, and in 2008 they were made available in North America as EndoSequence^®^ BC sealer/RRM/RRM-Fast Set Putty™, and recently in Europe as TotalFill^®^ BC sealer/RRM Paste/RRM Putty/Fast putty™ [19,20]. Even though these materials are sold under different brand names, they have the same composition, and physical and biological properties [21]. The premixed calcium silicate-based bioceramics materials are stain-free, ready to use without mixing, and exhibit comparable physical and chemical properties to MTA with better handling properties [19,20,21,22,23]. Recently, NeoPUTTY^®^ was introduced to the market as another premixed bioceramic that was approved by the U.S. Food and Drug Administration (FDA) in 2020.

Adequate bond strength between bioceramic materials and restorative materials is very crucial to the success rate of VPT [24]. A variety of restorative materials are used in VPT including zinc oxide–eugenol cement base (ZOE), IRM, resin-modified glass ionomer (RMGI), and resin composite (RC) [25,26,27,28]. The shear bond strength (SBS) between MTA and RC has been evaluated in multiple studies using different etching and bonding techniques, which resulted in a wide range of values [29,30,31,32,33,34,35,36,37,38]. The SBS of RC to premixed bioceramics has been investigated in limited studies and reported to be lower than MTA [34,39]. The literature is still ambiguous about the restorative protocol following the placement of bioceramic materials, as well as the optimal time for permanent restoration placement [40]. Few researchers have compared the SBS of RC and RMGI to bioceramic materials, and they found a stronger bond strength with RC [31,32,35]. The impact of RC restoration timing on the SBS was investigated by Palma et al., who found that a delayed restorative procedure 7 days following MTA resulted in a significantly higher SBS, while with premixed bioceramics no significant difference was found between immediate and delayed restorations [39,41].

To date, there is a lack of studies evaluating the physical and chemical properties of the newly introduced bioceramic materials such as NeoPUTTY and NeoMTA 2. Moreover, the SBS of different restorative materials to the new bioceramic materials has still not been fully investigated. Furthermore, the impact of immediate or delayed placement of different restorative materials on the SBS to the bioceramic materials needs to be assessed. Therefore, this study aimed to (1) evaluate the SBS of RC and RMGI to different bioceramic materials (NeoPUTTY^®^, NeoMTA2^®^, TotalFill^®^ BC RRM™ Fast Set Putty and ProRoot MTA^®^) and (2) compare the effects of immediate vs. delayed placement of restoration on the SBS. The null hypothesis was that there would be (1) no significant difference in the SBS of RC and RMGI to the four tested bioceramic materials; and (2) there would be no significant difference between immediate and delayed placement of restorations (RC and RMGI) on its SBS to the bioceramic materials.

## 2. Materials and Methods

### 2.1. Specimen Preparation

A total of 160 Teflon blocks with a central retentive hole 5 mm in diameter and 2 mm in height were prepared and randomly filled using a list of random numbers generated online with a randomization program (http://www.randomizer.org, accessed on 25 June 2021) with one of the 4 experimental groups (NeoPUTTY^®^, NeoMTA2^®^, TotalFill^®^ BC RRM™ Fast Set Putty, and ProRoot^®^ MTA), 40 blocks/material. Then each group was subdivided into two subgroups (20 blocks/group) that were either restored with RC (Filtek™ Z350 XT Flowable Restorative, 3M ESPE, St. Paul, MN, USA) or RMGI (GC Fuji II LC^®^ Capsule, GC America, IL, USA). Each subgroup was further divided into 10 blocks in which the restorative material was placed over the bioceramic materials either immediately or delayed after setting of bioceramic materials in a humidifier for 7 days (Table 1).

### 2.2. Materials Placement

The NeoMTA2^®^ and ProRoot^®^ MTA groups were mixed according to the manufacturer’s instructions (Table 2), then placed in the holes of the Teflon block using a spatula. The NeoPUTTY^®^ and TotalFill^®^ groups were injected directly into the Teflon block holes (Table 2). Then, the materials were condensed and flattened with a glass microscopic slide to ensure their good adaptation to the hole and to create a flat surface.

For the immediate restorative groups, a universal adhesive (Single bond universal 3M, ESPE, St. Paul, MN, USA) was applied above the bioceramic materials only for the RC groups, and then it was rubbed for 20 s, air-dried for 5 s, then light-cured for 10 s. A putty mold with a central hole 3 mm in diameter and 2 mm in height was then centered above the bioceramic materials and either RC Filtek™ Z350 XT Flowable Restorative shade A2 or RMGI GC Fuji II LC^®^ Capsule shade A2 was injected and covered with a Mylar^®^ strip (Mylar Uni-Strip, Caulk/Dentsply, Milford, DE, USA) and pressed with a glass microscopic slide (200 Milligrams) to flatten the surface and remove any voids. The material was light-cured for 20 s using a single-wave light cure unit with a light intensity of 1200 mW/cm^2^ (Elipar S10, 3M™ ESPE™, St. Paul, MN, USA) with the curing tip perpendicular to the mold surface and centered directly over the opening.

For the delayed restorative groups, the Teflon blocks containing the bioceramic materials were stored in an incubator (GI2 So-Low Cincinnati, OH, USA) at a temperature of 37 °C and 100% humidity for 7 days to ensure the complete setting of the bioceramic materials [39]. Then, the restorative materials were placed in the manner explained previously for the immediate groups.

After completion of the restorative procedure, all specimens were stored in an incubator for a period of 72 h at a temperature of 37 °C with 100% humidity prior to testing the shear bond strength.

### 2.3. Shear Bond Strength Test

**Figure 1:**
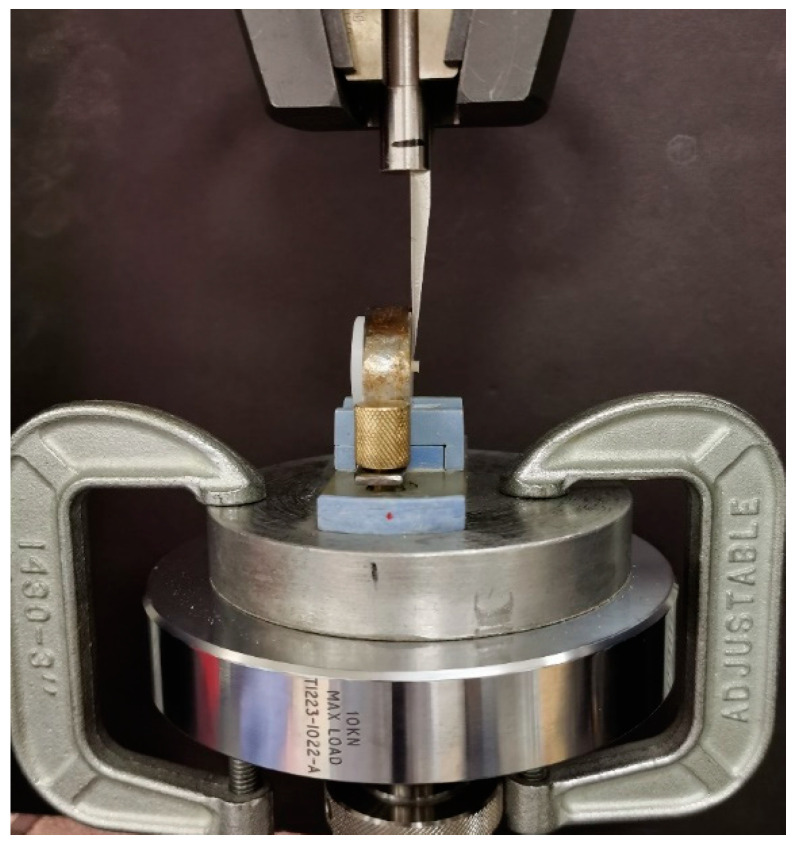
A sample loaded in the universal testing machine.

The specimens were mounted in the universal testing machine (Instron, model no. 8500, Illinois Tool Works Inc., Norwood, MA, USA) with the crosshead perpendicular and flush with the interface of the restoration and the bioceramic material (Figure 1). The specimens were loaded at a crosshead speed of 1 mm/min using a knife-edge blade. The SBS was calculated as megapascals (MPa) using the following formula: stress (MPa) = force (N)/bonding area (mm^2^).

### 2.4. Fracture Analysis

The specimens and fractured surfaces were examined using the Digital Microscope KH-7700 (Hirox Europe Ltd., Limonest, France) at 40× magnification by one blinded independent examiner to determine the mode of failure. The modes of failure were classified as follows: (A) cohesive failure within the restorative material, (B) cohesive failure within the bioceramic material, (C) adhesive failure at the interface between the bioceramic and restorative materials, or (D) mixed failure (a combination of adhesive and cohesive failure) [42].

### 2.5. Sample Size Calculation and Statistical Analysis

The sample size was calculated using the G*Power program (Version 3.1.9.4) at α = 0.05 with an effect size of 0.35 and power: 0.9; the total sample size was at least 160 (10 block/group). Data were analyzed using SPSS version 24 statistical software (IBM Inc., Chicago, IL, USA). The shear bond strength results are described using mean and standard deviation (SD). The mean shear bond strengths of the groups were compared using one-way analysis of variance (ANOVA) and the Games–Howell post hoc test was used to detect statistically significant differences between the bioceramic groups. The independent t test was used to detect the differences between the restorative materials (RC vs. RMGI) and between the restorative timings (immediate vs. delayed). Statistical significance was established as *p* ≤ 0.05.

## 3. Results

The mean SBS of the immediately placed RC and RMGI to the bioceramic materials are presented in Table 3. Overall, the SBS of RC to each bioceramic material was significantly higher than to RMGI except for ProRoot MTA (*p*-value 0.65). In the RC groups, NeoMTA2 and ProRoot MTA showed the highest mean SBS (8.57 and 7.64 MPa, respectively), which was significantly higher than both NeoPUTTY and TotalFill^®^ BC RRM™ Fast Set Putty (4.04 and 4.38 MPa, respectively). In the RMGI groups, the mean SBS of ProRoot MTA (7.18 MPa) was significantly higher than the other materials, followed by NeoMTA2 (4.15 MPa), which was also significantly higher than NeoPUTTY and TotalFill^®^ BC RRM™ Fast Set Putty (1.62 and 1.54 MPa, respectively).

The SBS of the delayed RC groups for all bioceramic materials was significantly higher than immediate groups. Similarly, the SBS of delayed RMGI groups with NeoPUTTY and TotalFill^®^ BC RRM™ Fast Set Putty was significantly higher than that of immediate groups, whereas the SBS of the immediate RMGI placement over ProRoot and NeoMTA2 was statistically higher than that of the delayed timing (Table 4).

**Figure 2:**
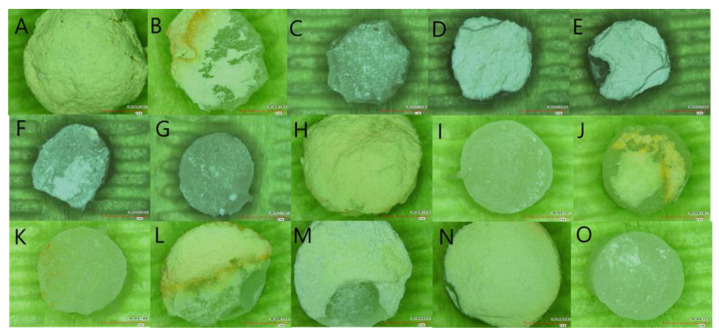
Photographs of samples representing fracture mode under Digital Microscope at 40× magnification. (**A**) NeoMTA2 cohesive fracture. (**B**) NeoMTA2 Mixed fracture. (**C**) NeoMTA2 adhesive fracture. (**D**) NeoPUTTY cohesive fracture. (**E**) NeoPUTTY mixed fracture. (**F**) NeoPUTTY mixed fracture. (**G**) NeoPUTTY adhesive. (**H**) ProRootMTA cohesive fracture. (**I**) ProRootMTA adhesive fracture. (**J**) ProRootMTA mixed fracture. (**K**) ProRootMTA adhesive fracture. (**L**) ProRootMTA mixed fracture. (**M**) TotalFill mixed fracture. (**N**) TotalFill cohesive fracture. (**O**) TotalFill adhesive fracture.

Table 5 shows the fracture type for the experimental groups. Overall, there was no fracture within the restorative materials. Fractures occurred at the bioceramic material (43.75%), at the interface between bioceramic material and the restoration (12.5%), or at a combination of the two (43.75%) (Figure 2).

## 4. Discussion

The bond strength between bioceramic materials used in VPT and restorative materials is very important and plays an integral role in providing a good seal and ultimately in treatment success and prognosis [24]. In this study, the SBS of RC and RMGI to new commercially available bioceramic materials was evaluated. Most studies evaluating SBS allow the bioceramic materials to set completely or initially for at least 45 min before placing the restorative materials, which does not mimic the clinical situation. Thereby, in this study, the SBS of restorative materials was evaluated immediately following the placement of the bioceramic materials, which represented the single visit restorative scenario. No acid etching or conditioning was included in this study for two reasons: first, to prevent bioceramic material washout, and second, not to interfere with the bioceramic material’s physical properties, as it was found that the acid etching of newly mixed MTA reduces the compressive strength and surface microhardness [43].

Based on the results obtained from the present study, the null hypothesis was rejected as there was a significant difference in the SBS between restorative materials and different bioceramics, and restorative timings. In this study, the SBS of RC to the tested bioceramic materials in the immediately restored groups was significantly higher than RMGI except with ProRoot MTA. This finding agreed with previous studies, which showed a higher SBS of RC in comparison to RMGI [31,32,35]. This might be attributed to the use of a bonding agent with RC and not with RMGI. In addition, in this study the SBS of the MTA groups to RC and RMGI was significantly higher than that of the premixed bioceramics groups, which is in agreement with Hursh et al., where NeoMTA and ProRoot MTA had a higher SBS value to RC than the premixed EndoSequence RRM [34].

In the literature, few studies have assessed the SBS of the bioceramic materials to immediately placed RC or RMGI [39,41,44]. Palma et al. found the SBS of RC to ProRoot MTA and TotalFill to be 1.33 and 1.22 MPa, respectively [39,41]. Similarly, the SBS of RC to NeoPUTTY was reported to be 1.6 MPa [44]. These SBS values were lower than the values reported in this study, possibly because of their use of different flowable resin restorative materials such as SDR Bulk fill flowable composite [39,41] and self-adhering flowable composite [44], both of which have a different composition from Filtek™ Z350 XT flowable composite. Regarding NeoMTA2, there were no available data in the literature to compare it with. Generally, there are few studies evaluating the SBS of bioceramic materials to immediately placed RMGI; Al-homaidhi‘s study evaluated the SBS of immediately placed RMGI to NeoPUTTY and reported a mean SBS value of 0.68 MPa, which was comparable to this study result [44].

It is generally advised that the restorative procedure should be delayed between 72 and 96 h following MTA mixing to enable the material to achieve its optimum physical properties, as etching, rinsing, priming, and condensation pressure could affect the setting of MTA [2,3,45]. Few researchers have evaluated the effect of restoration timing on the SBS of bioceramic materials [39,41,44]. Palma et al. found that it was preferable to delay the RC restorative procedure after ProRoot MTA placement as it had a statistically higher SBS than immediate placement [41]. Al-homaidhi reported a higher SBS of NeoPUTTY with delayed RC and RMGI restorations in comparison with immediate restoration [44]. These findings correlated with the current study results, where delayed restoration timing of the tested bioceramic materials had higher SBS values than immediate restoration, except for MTA groups with RMGI. The hydrophobic nature of the RC and the presence of acidic monomers (MDP Phosphate Monomer) in the universal adhesive system which causes etching of the newly formed crystallin structure and the gel-like amorphous layer of the bioceramic material could be related to the lower SBS in the immediate RC groups [46,47]. However, Palma et al. in another recent study presented opposing results when they found that there was no significant difference between immediate and delayed RC restoration placement with premixed bioceramics (TotalFill RRM); however, their SBS values between immediate and delayed RC restoration need to be considered (1.22 and 5.22 MPa, respectively) [39].

Immediate placement of RMGI on ProRoot MTA and NeoMTA2 showed a statistically higher SBS than the delayed placement. The relatively high bond strength between the unset MTA and RMGI could be related to the formation of a chemical bond between the carboxylate anion (RCOO−) in the polyacrylic acid and the calcium in the MTA during setting [48], which was supported by the predominant cohesive or mixed failure within the MTA groups rather than adhesive failure. This finding indicates that RMGI could be placed immediately above MTA in a single visit procedure. However, the premixed bioceramics did not react similarly to MTA with immediately placed RMGI, as they need moisture from external sources to start the setting reaction, unlike the MTA, where the setting reaction begins immediately following the mixing of powder and liquid.

In this study, the mean SBS of ProRoot MTA with delayed RC placement was comparable to the previous investigations [30,31,32,34,35]. However, it was lower than in Alhowaish et al., where they reported the SBS of ProRoot MTA to be 22.11 MPa; this high value could be due to the different methodology used in their study, where they used cylindrical metal molds to fabricate disc specimens as well as 35% phosphoric acid etching and the application of two consecutive coats of the adhesive system [33]. In this study the mean SBS of NeoMTA2 with delayed RC placement was comparable with the mean SBS of NeoMTA to delayed RC in Hursh et al.’s study (5.72 MPa) [34]. Furthermore, the SBS of delayed RC to premixed bioceramics in the current study was higher than in the previous investigation [39,44]. However, the SBS of delayed RMGI to NeoPUTTY recorded in this study was comparable to the 2.88 MPa that was reported in a previous study [44].

Concerning the failure modes, the examination demonstrated that the two most common failure modes among all tested bioceramic materials were cohesive within the bioceramic or a mixed cohesive–adhesive fracture, and no specimen showed cohesive failure within the restorative material. This result correlates with previously conducted studies on MTA and premixed bioceramics with more cohesive and mixed failures [34,39,41,44]. The bonding strength is considered acceptable when the failure is cohesive within the bioceramic material, which indicates strong adhesion between the bioceramic materials and restoration. Both MTA and premixed bioceramics groups exhibited cohesive/mixed failures regardless of the difference in their SBS values, which could stem from different intrinsic cohesive strengths of the tested bioceramic materials.

In VPT, restorative and bioceramic materials bond with each other and with dentine. In this study, the bond strength was only evaluated between the bioceramic and restorative materials without considering the bonding to dentin. In the clinical situation where dentin is present, there are three interfaces: bioceramic/dentin, restorative material/dentin, and bioceramic/restorative material. Only the third interface (bioceramic/restorative material) was evaluated in this study; therefore, the results need to be interpreted and adapted to the clinical context with caution. For the bioceramic/dentin interface, the exact bonding mechanism is not fully clear; after placing the bioceramic material on dentine hydroxyapatite crystals form and fill the microscopic gaps leading to a chemical bond between the two surfaces [49]. The restorative material/dentin interface forms a rim around the other two surfaces, and it has been reported that a minimum dentin bond strength of 17–20 MPa is needed to resist the contraction forces of polymerization shrinkage and to achieve a properly sealed gap-free restoration [50,51,52]. This value is higher than the bond strength values between restorative materials and bioceramic materials shown in this study which further improve the total seal of the VPT. This might imply that the bond strength between the restoration and dentin is more important, emphasizing the need for proper techniques and meticulous isolation to optimize the bond strength between restoration and dentin. Moreover, in the present study no acid etching was used in the delayed RC groups in order to standardize the study methodology with the immediate groups. However, a recent systematic review and meta-analysis found that bond strength of RC to MTA was higher with a total-etch adhesive strategy as the acid etching improves the bond strength between RC and MTA [53]. Another limitation of this study is that the mode of failure was only assessed using a digital microscope; the use of Scanning Electron Microscopy (SEM) analysis to further evaluate the fracture interfaces would be recommended.

This study provides baseline information for the SBS of RC and RMGI to four different bioceramics used in VPT. Additionally, it examined the effect of restoration type and timing on the SBS. Nevertheless, for more improved insights and to further mimic the clinical situation, more research studies are desirable to represent the clinical situation by considering the bonding to dentin in extracted teeth. Moreover, more research should be conducted to investigate how to improve the SBS of immediate restorations and the possibility of using bonding agents with RMGI to improve its bond strength. Moreover, the clinical performance of these newly introduced bioceramic materials in VPT should be investigated. On the biological level, MTA is known to enhance the osteogenic differentiation of dental pulp stem cells; however, the role of premixed bioceramic materials in association with stem cells and their osteogenic stimuli for undifferentiated cells should be investigated [54,55].

## 5. Conclusions

Based on the limitations of this study, the following conclusions can be made:

- The mean SBS of RC to the bioceramic materials was significantly higher than RMGI except for ProRoot MTA;
- The mean SBS of RC and RMGI to MTA groups was significantly higher than that of premixed bioceramics;
- The SBS of delayed RC was significantly higher than that of immediate timing in all bioceramic materials;
- The SBS of delayed RMGI was significantly higher than immediate timing in the premixed bioceramic groups, whereas the SBS of immediate RMGI placement over MTA was statistically higher than that of delayed timing.

## Figures and Tables

**Table 1:** Distribution of experimental groups.

Bioceramic Materials	Restorative Materials			
	Filtek™ Z350 XT Flowable		GC Fuji II LC^®^	
	Immediate	Delayed	Immediate	Delayed
ProRoot^®^ MTA (n = 40)	10	10	10	10
NeoMTA 2^®^ (n = 40)	10	10	10	10
TotalFill^®^ BC RRM™ Fast Set Putty (n = 40)	10	10	10	10
NeoPUTTY^®^ (n = 40)	10	10	10	10
Total = 160	40	40	40	40
	80		80	

**Table 2:** Chemical composition and application procedure of bioceramic materials according to the manufacturer’s instructions.

Bioceramic	Manufacturer	Composition	Application
ProRoot^®^ MTA (Mineral Trioxide Aggregate)	DENTSPLY, Tulsa, OK, USA	Bismuth oxide, tricalcium silicate, dicalcium silicate, calcium dialuminate, and calcium sulfate dehydrated	Mixed powder/liquid ratio: 1/3
NeoMTA 2^®^ (Mineral Trioxide Aggregate)	Nusmile Inc., Houston, TX; USA	Powder and gel system consisting of an extremely fine, inorganic powder of tricalcium and dicalcium silicate, which is mixed with the water-based gel	Mix 1 scoop (0.05 gm or 0.1 gm) of powder with one or two drops of gel.
TotalFill^®^ BC RRM™ Fast Set Putty	FKG Dentaire SA, La Chaux-de-Fonds, Switzerland	Calcium silicate, zirconium oxide, tantalum oxide, calcium phosphate monobasic, and fillers	Pre-mixed material and ready to apply
NeoPUTTY^®^	Nusmile Inc., Houston, TX; USA	Bioactive paste consisting of an extremely fine, inorganic powder of tricalcium/dicalcium silicate in an organic medium	Pre-mixed material and ready to apply

**Table 3:** Shear bond strength of different bioceramics with immediately placed Filtek™ Z350 XT flowable composite and GC Fuji II LC^®^ resin modified glass ionomer in megapascals (MPa).

Restoration		Bioceramics				*p*-Value *
		ProRoot MTA	NeoMTA2	TotalFill	NeoPUTTY	
**Filtek™ Z350 XT**	Mean ± SD	7.64 ± 1.82 a	8.57 ± 1.84 a	4.38 ± 0.65 b	4.04 ± 0.93 b	<0.001
**GC Fuji II LC^®^**	Mean ± SD	7.18 ± 2.60 a	4.15 ± 0.35 b	1.54 ± 0.18 c	1.62 ± 0.12 c	<0.001
***p*** **-value** **		0.655	0.001	<0.001	0.002	

Different lowercase superscript letters within one raw indicate statistical significance (*p* < 0.05) as measured by Games–Howell post hoc test. * One-way analysis of variance (ANOVA) ** Independent t-test.

**Table 4:** Comparison of mean± standard deviation (SD) of immediate vs. delayed restoration with bioceramic materials.

Restoration	Bioceramic	Immediate (Mean ± SD)	Delayed (Mean ± SD)	*p-*Value
**Filtek** **™ Z350 XT**	ProRoot MTA	7.64 ± 1.82	14.65 ± 0.95	<0.001
	NeoMTA2	8.57 ± 1.84	12.01 ± 3.01	0.006
	TotalFill	4.38 ± 0.65	13.66 ± 3.81	0.002
	NeoPUTTY	4.04 ± 0.93	8.03 ± 1.82	<0.001
**GC Fuji II LC^®^**	ProRoot MTA	7.18 ± 2.60	3.33 ± 1.12	0.001
	NeoMTA2	4.15 ± 0.35	2.31 ± 1.35	0.002
	TotalFill	1.54 ± 0.18	2.57 ± 0.23	<0.001
	NeoPUTTY	1.62 ± 0.12	3.27 ± 0.44	<0.001

**Table 5:** Fracture mode of the groups after shear bond strength testing.

Bioceramic	Restoration	Timing	Failure Type		
			Cohesive within Bioceramic	Adhesive	Mixed
**ProRoot MTA**	Filtek™ Z350 XT	Immediate	80% (8/10)	0	20% (2/10)
		Delayed	50% (5/10)	0	50% (5/10)
	GC Fuji II LC^®^	Immediate	20% (2/10)	20% (2/10)	60% (6/10)
		Delayed	10% (1/10)	0	60% (6/10)
**NeoMTA2^®^**	Filtek™ Z350 XT	Immediate	30% (3/10)	20% (2/10)	70% (5/10)
		Delayed	50% (5/10)	0	50% (5/10)
	GC Fuji II LC^®^	Immediate	60% (6/10)	0	40% (4/10)
		Delayed	10% (1/10)	10% (1/10)	80% (8/10)
**TotalFill** **^®^ BC RRM™ Fast Set Putty**	Filtek™ Z350 XT	Immediate	40% (4/10)	0	60% (6/10)
		Delayed	100% (10/10)	0	0
	GC Fuji II LC^®^	Immediate	0	40% (4/10)	60% (6/10)
		Delayed	10% (1/10)	40% (4/10)	50% (5/10)
**NeoPUTTY** **^®^**	Filtek™ Z350 XT	Immediate	80% (8/10)	0	20% (2/10)
		Delayed	70% (7/10)	0	30% (3/10)
	GC Fuji II LC^®^	Immediate	90% (9/10)	0	10% (1/10)
		Delayed	20% (2/10)	40% (4/10)	40% (4/10)

## Data Availability

The data presented in this study are available on request from the corresponding author.

## References

[1] Davaie S., Hooshmand T., Ansarifard S. (2021). Different Types of Bioceramics as Dental Pulp Capping Materials: A Systematic Review. Ceram. Int..

[2] Parirokh M., Torabinejad M., Dummer P.M.H. (2018). Mineral Trioxide Aggregate and Other Bioactive Endodontic Cements: An Updated Overview—Part I: Vital Pulp Therapy. Int. Endod. J..

[3] Torabinejad M., Parirokh M., Dummer P.M.H. (2018). Mineral Trioxide Aggregate and Other Bioactive Endodontic Cements: An Updated Overview—Part II: Other Clinical Applications and Complications. Int. Endod. J..

[4] Lin P.Y., Chen H.S., Wang Y.H., Tu Y.K. (2014). Primary Molar Pulpotomy: A Systematic Review and Network Meta-Analysis. J. Dent..

[5] Shirvani A., Asgary S. (2014). Mineral Trioxide Aggregate versus Formocresol Pulpotomy: A Systematic Review and Meta-Analysis of Randomized Clinical Trials. Clin. Oral Investig..

[6] Coll J.A., Seale N.S., Vargas K., Marghalani A.A., Al Shamali S., Graham L. (2017). Primary Tooth Vital Pulp Therapy: A Systematic Review and Meta-Analysis. Pediatr. Dent..

[7] Marghalani A.A., Omar S., Chen J.W. (2014). Clinical and Radiographic Success of Mineral Trioxide Aggregate Compared with Formocresol as a Pulpotomy Treatment in Primary Molars: A Systematic Review and Meta-Analysis. J. Am. Dent. Assoc..

[8] Asgary S., Shirvani A., Fazlyab M. (2014). MTA and Ferric Sulfate in Pulpotomy Outcomes of Primary Molars: A Systematic Review and Meta-Analysis. J. Clin. Pediatr. Dent..

[9] Camilleri J. (2014). Color Stability of White Mineral Trioxide Aggregate in Contact with Hypochlorite Solution. J. Endod..

[10] Marciano M.A., Duarte M.A.H., Camilleri J. (2015). Dental Discoloration Caused by Bismuth Oxide in MTA in the Presence of Sodium Hypochlorite. Clin. Oral Investig..

[11] Parirokh M., Torabinejad M. (2010). Mineral Trioxide Aggregate: A Comprehensive Literature Review-Part I: Chemical, Physical, and Antibacterial Properties. J. Endod..

[12] Watts J.D., Holt D.M., Beeson T.J., Kirkpatrick T.C., Rutledge R.E. (2007). Effects of PH and Mixing Agents on the Temporal Setting of Tooth-Colored and Gray Mineral Trioxide Aggregate. J. Endod..

[13] Boutsioukis C., Noula G., Lambrianidis T. (2008). Ex Vivo Study of the Efficiency of Two Techniques for the Removal of Mineral Trioxide Aggregate Used as a Root Canal Filling Material. J. Endod..

[14] Eren S.K., Örs S.A., Aksel H., Canay Ş., Karasan D. (2022). Effect of Irrigants on the Color Stability, Solubility, and Surface Characteristics of Calcium-Silicate Based Cements. Restor. Dent. Endod..

[15] Metlerska J., Fagogeni I., Metlerski M., Nowicka A. (2021). Vital Pulp Therapy in Aesthetic Zone-Identifying the Biomaterial That Reduces the Risk of Tooth Discolouration. Materials.

[16] Camilleri J. (2015). Staining Potential of Neo MTA Plus, MTA Plus, and Biodentine Used for Pulpotomy Procedures. J. Endod..

[17] Alsanouni M., Bawazir O.A. (2019). A Randomized Clinical Trial of NeoMTA Plus in Primary Molar Pulpotomies. Pediatr. Dent..

[18] Keskin C., Sarıyılmaz E. (2018). Color Stability of NeoMTA Plus and MTA Plus When Mixed with Anti-Washout Gel or Distilled Water. Meandros Med. Dent. J..

[19] Ree M., Schwartz R. (2014). Clinical Applications of Bioceramic Materials in Endodontics Endodontic Practice US. Endod. Pract..

[20] Motwani N., Ikhar A., Nikhade P., Chandak M., Rathi S., Dugar M., Rajnekar R. (2021). Premixed Bioceramics: A Novel Pulp Capping Agent. J. Conserv. Dent..

[21] Debelian G., Trope M. (2016). ScienceDirect the Use of Premixed Bioceramic Materials in Endodontics. G. Ital. Endod..

[22] Malhotra S. (2014). Bioceramic Technology in Endodontics. Br. J. Med. Med. Res..

[23] El-Sherif S.M., Sherief D.I., El-Refai D.A. (2022). Evaluation of the PH, Calcium Ion Release, and Antibacterial Effect of a Premixed Bioceramic Endodontic Sealer. Gen. Dent..

[24] van Meerbeek B., Peumans M., Poitevin A., Mine A., van Ende A., Neves A., de Munck J. (2010). Relationship between Bond-Strength Tests and Clinical Outcomes. Dent. Mater..

[25] Keswani D., Pandey R.K., Ansari A., Gupta S. (2014). Comparative Evaluation of Platelet-Rich Fibrin and Mineral Trioxide Aggregate as Pulpotomy Agents in Permanent Teeth with Incomplete Root Development: A Randomized Controlled Trial. J. Endod..

[26] Eid A., Mancino D., Rekab M.S., Haikel Y., Kharouf N. (2022). Effectiveness of Three Agents in Pulpotomy Treatment of Permanent Molars with Incomplete Root Development: A Randomized Controlled Trial. Healthcare.

[27] Careddu R., Duncan H.F. (2021). A Prospective Clinical Study Investigating the Effectiveness of Partial Pulpotomy after Relating Preoperative Symptoms to a New and Established Classification of Pulpitis. Int Endod. J..

[28] Abuelniel G.M., Duggal M.S., Kabel N. (2020). A Comparison of MTA and Biodentine as Medicaments for Pulpotomy in Traumatized Anterior Immature Permanent Teeth: A Randomized Clinical Trial. Dent. Traumatol. Off. Publ. Int. Assoc. Dent. Traumatol..

[29] Tunç E.Ş., Sönmez I.S., Bayrak Ş., Eǧilmez T. (2008). The Evaluation of Bond Strength of a Composite and a Compomer to White Mineral Trioxide Aggregate with Two Different Bonding Systems. J. Endod..

[30] Sindi A.S. (2021). An In Vitro Study to Assess the Effectiveness of the Shear Bond Strength of Mineral Trioxide Aggregate with Different Adhesive Systems. J. Pharm. Bioallied Sci..

[31] Tulumbaci F., Almaz M.E., Arikan V., Mutluay M.S. (2017). Shear Bond Strength of Different Restorative Materials to Mineral Trioxide Aggregate and Biodentine. J. Conserv. Dent..

[32] Doozaneh M., Koohpeima F., Firouzmandi M., Abbassiyan F. (2017). Shear Bond Strength of Self-Adhering Flowable Composite and Resin-Modified Glass Ionomer to Two Pulp Capping Materials. Iran. Endod. J..

[33] Alhowaish L., Salama F., Al-Harbi M., Abumoatti M. (2020). Shear Bond Strength of a Resin Composite to Six Pulp Capping Materials Used in Primary Teeth. J. Clin. Pediatr. Dent..

[34] Hursh K.A., Kirkpatrick T.C., Cardon J.W., Brewster J.A., Black S.W., Himel V.T., Sabey K.A. (2019). Shear Bond Comparison between 4 Bioceramic Materials and Dual-Cure Composite Resin. J. Endod..

[35] Ajami A.A., Jafari Navimipour E., Savadi Oskoee S., Abed Kahnamoui M., Lotfi M., Daneshpooy M. (2013). Comparison of Shear Bond Strength of Resin-Modified Glass Ionomer and Composite Resin to Three Pulp Capping Agents. J. Dent. Res. Dent. Clin. Dent. Prospect..

[36] Oskoee S.S., Kimyai S., Bahari M., Eghbal P.M.M.J., Asgary S. (2011). Comparison of Shear Bond Strength of Calcium-Enriched Mixture Cement and Mineral Trioxide Aggregate to Composite Resin. J. Contemp. Dent. Pract..

[37] Nagi S.M., Omar N., Salem H.N., Aly Y. (2020). Effect of Different Surface Treatment Protocols on the Shear Bond Strength of Perforation Repair Materials to Resin Composite. J. Adhes. Sci. Technol..

[38] Samimi P., Kazemian M., Shirban F., Alaei S., Khoroushi M. (2018). Bond Strength of Composite Resin to White Mineral Trioxide Aggregate: Effect of Different Surface Treatments. J. Conserv. Dent..

[39] Palma P.J., Marques J.A., Antunes M., Falacho R.I., Sequeira D., Roseiro L., Santos J.M., Ramos J.C. (2020). Effect of Restorative Timing on Shear Bond Strength of Composite Resin/Calcium Silicate–Based Cements Adhesive Interfaces. Clin. Oral Investig..

[40] Tsujimoto M., Tsujimoto Y., Ookubo A., Shiraishi T., Watanabe I., Yamada S., Hayashi Y. (2013). Timing for Composite Resin Placement on Mineral Trioxide Aggregate. J. Endod..

[41] Palma P.J., Marques J.A., Falacho R.I., Vinagre A., Santos J.M., Ramos J.C. (2018). Does Delayed Restoration Improve Shear Bond Strength of Different Restorative Protocols to Calcium Silicate-Based Cements?. Materials.

[42] von Fraunhofer J.A. (2012). Adhesion and Cohesion. Int J. Dent..

[43] Kayahan M.B., Nekoofar M.H., Kazandağ M., Canpolat C., Malkondu O., Kaptan F., Dummer P.M.H. (2009). Effect of Acid-Etching Procedure on Selected Physical Properties of Mineral Trioxide Aggregate. Int. Endod. J..

[44] Al-Homaidhi M. (2021). Shear Bond Strength of an Endodontic Tricalcium Silicate- Based Putty to Different Adhesive Systems at Different Time Intervals. J. Res. Med. Dent. Sci..

[45] Torabinejad M., Chivian N. (1999). Clinical Applications of Mineral Trioxide Aggregate. J. Endod..

[46] Hidari T., Takamizawa T., Imai A., Hirokane E., Ishii R., Tsujimoto A., Suzuki T., Miyazaki M. (2020). Role of the Functional Monomer 10-Methacryloyloxydecyl Dihydrogen Phosphate in Dentin Bond Durability of Universal Adhesives in Etch-&-Rinse Mode. Dent. Mater. J..

[47] Chang S.-W. (2012). Chemical Characteristics of Mineral Trioxide Aggregate and Its Hydration Reaction. Restor. Dent. Endod..

[48] Eid A.A., Komabayashi T., Watanabe E., Shiraishi T., Watanabe I. (2012). Characterization of the Mineral Trioxide Aggregate-Resin Modified Glass Ionomer Cement Interface in Different Setting Conditions. J. Endod..

[49] Kaup M., Dammann C.H., Schäfer E., Dammaschke T. (2015). Shear Bond Strength of Biodentine, ProRoot MTA, Glass Ionomer Cement and Composite Resin on Human Dentine Ex Vivo. Head Face Med..

[50] Davidson C.L., de Gee A.J., Feilzer A. (1984). The Competition between the Composite-Dentin Bond Strength and the Polymerization Contraction Stress. J. Dent. Res..

[51] Teixeira C.S., Chain M.C. (2010). Evaluation of Shear Bond Strength between Self-Etching Adhesive Systems and Dentin and Analysis of the Resin-Dentin Interface. Gen. Dent..

[52] Triolo P.T.J., Swift E.J.J., Barkmeier W.W. (1995). Shear Bond Strengths of Composite to Dentin Using Six Dental Adhesive Systems. Oper. Dent..

[53] Hardan L., Mancino D., Bourgi R., Alvarado-Orozco A., Rodríguez-Vilchis L.E., Flores-Ledesma A., Cuevas-Suárez C.E., Lukomska-Szymanska M., Eid A., Danhache M.-L. (2022). Bond Strength of Adhesive Systems to Calcium Silicate-Based Materials: A Systematic Review and Meta-Analysis of In Vitro Studies. Gels.

[54] Ballini A., Mastrangelo F., Gastaldi G., Tettamanti L., Bukvic N., Cantore S., Cocco T., Saini R., Desiate A., Gherlone E. (2015). Osteogenic Differentiation and Gene Expression of Dental Pulp Stem Cells under Low-Level Laser Irradiation: A Good Promise for Tissue Engineering. J. Biol. Regul. Homeost. Agents.

[55] Tetè G., D’Orto B., Nagni M., Agostinacchio M., Polizzi E., Agliardi E. (2020). Role of Induced Pluripotent Stem Cells (IPSCS) in Bone Tissue Regeneration in Dentistry: A Narrative Review. J. Biol. Regul. Homeost. Agents.

